# Juvenile xanthogranuloma as a potential early manifestation of neurofibromatosis type 1: A case report

**DOI:** 10.1097/MD.0000000000044223

**Published:** 2025-09-26

**Authors:** Lingxi Liu, Chunyan Mao

**Affiliations:** a Department of Dermatology, West China Second University Hospital, Sichuan University, Key Laboratory of Birth Defects and Related Diseases of Women and Children (Sichuan University), Ministry of Education, Chengdu, Sichuan Province, China.

**Keywords:** CALMs, juvenile xanthogranuloma, neurofibromatosis type 1, non-Langerhans cell histiocytoses

## Abstract

**Rationale:**

Juvenile xanthogranuloma (JXG), the most common form of non-Langerhans cell histiocytosis, typically presents in early childhood and often resolves spontaneously. Due to its generally favorable prognosis, physical examination may be overlooked. However, JXG has increasingly been associated with neurofibromatosis type 1 (NF1), highlighting the need for comprehensive evaluation.

**Patient concern:**

The patients were 14-month-old twin toddlers who presented to the outpatient clinic with multiple yellow papules on the scalp, first noted 2 months prior.

**Diagnosis:**

Physical examination revealed multiple yellow papules on the scalp, with dermoscopy showing the characteristic “setting sun” sign. Additionally, 10 to 20 café-au-lait macules of varying sizes were observed on the limbs and trunk. Genetic testing confirmed a mutation in the NF1 gene. Based on these findings, a final diagnosis of JXG associated with NF1 was made.

**Interventions:**

Comprehensive tests, including complete blood count, liver and kidney function tests, lipid profile, ophthalmologic screening, and brain magnetic resonance imaging, were performed on both patients. All results were unremarkable. Given the self-limiting nature of scalp JXG, no specific treatment was initiated, and regular follow-up was arranged.

**Outcomes:**

At 1-year follow-up, one twin developed a biopsy-confirmed plexiform neurofibroma and is scheduled to begin selumetinib therapy. The other twin remains asymptomatic under close monitoring.

**Lessons:**

Patients with JXG, especially those under the age of 2, should undergo a thorough physical examination to evaluate for NF1.

## 1. Introduction

Juvenile xanthogranuloma (JXG) is the most common form of non-Langerhans cell histiocytosis, typically presenting in early childhood as asymptomatic yellow-brown papules, often localized to the head and neck. It typically resolves spontaneously and has a favorable prognosis.^[1]^ Neurofibromatosis type 1 (NF1) is an autosomal dominant genetic disorder caused by mutations in the NF1 gene. Its clinical features include café-au-lait macules (CALMs), neurofibromas, and bone dysplasia. The estimated prevalence of NF1 is 1 in 3000 individuals, with clinical manifestations that may be present at birth or progressively develop with age.^[2,3]^ Recent studies suggest a potential association between JXG and NF1, indicating that JXG may serve as an early clinical manifestation of NF1.^[4–7]^ Although JXG generally has a good prognosis, its association with NF1 raises the importance of considering the coexistence of both conditions, especially in terms of early recognition and monitoring of NF1.

We report a case of twin infants diagnosed with JXG in conjunction with NF1, providing further evidence of the association between these 2 conditions. This case highlights the importance of considering NF1 in the early diagnosis and management of JXG.

## 2. Case presentation

**Figure 1.**
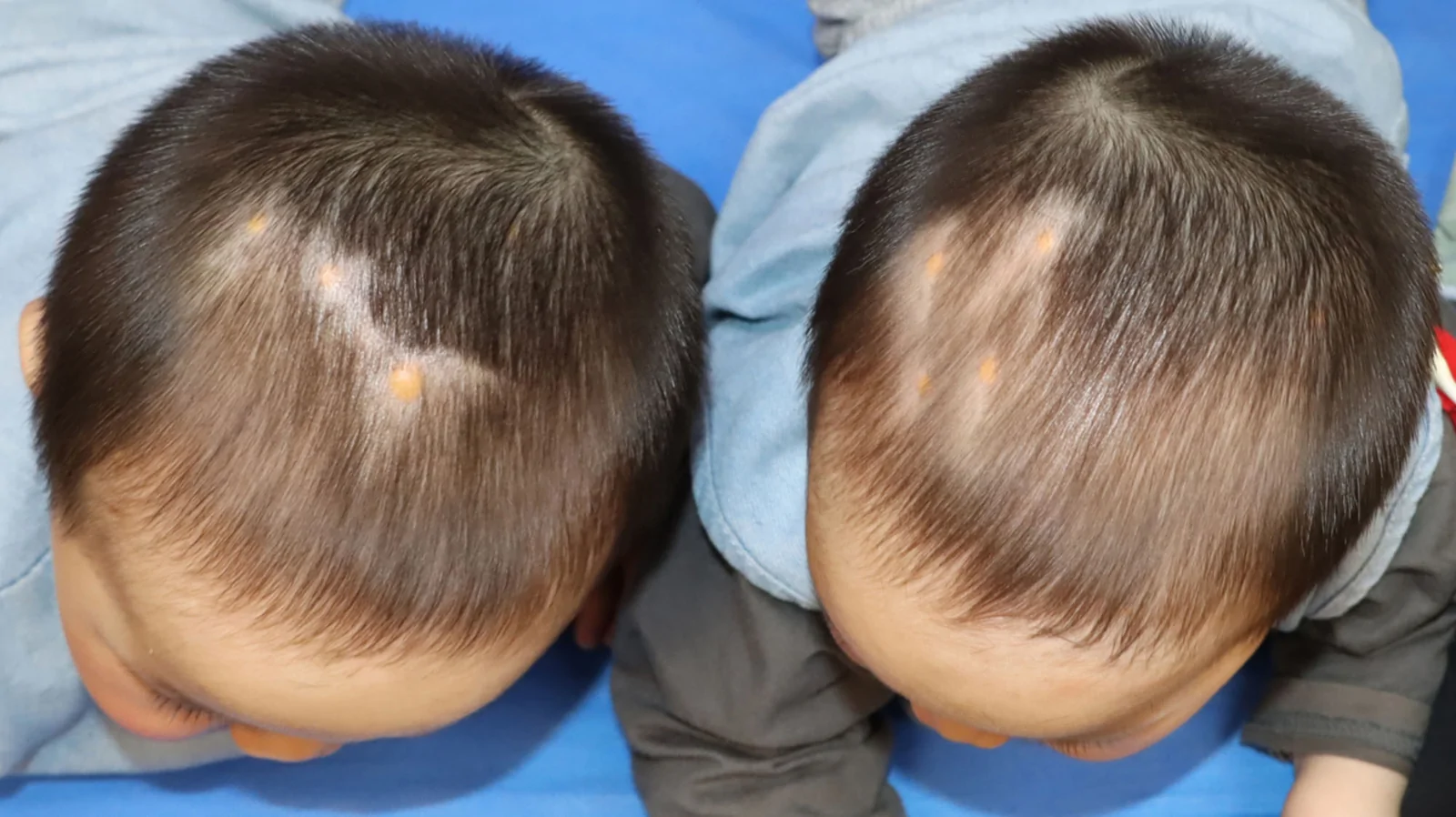
Multiple yellow papules on the scalp of monozygotic twins.

**Figure 2.**
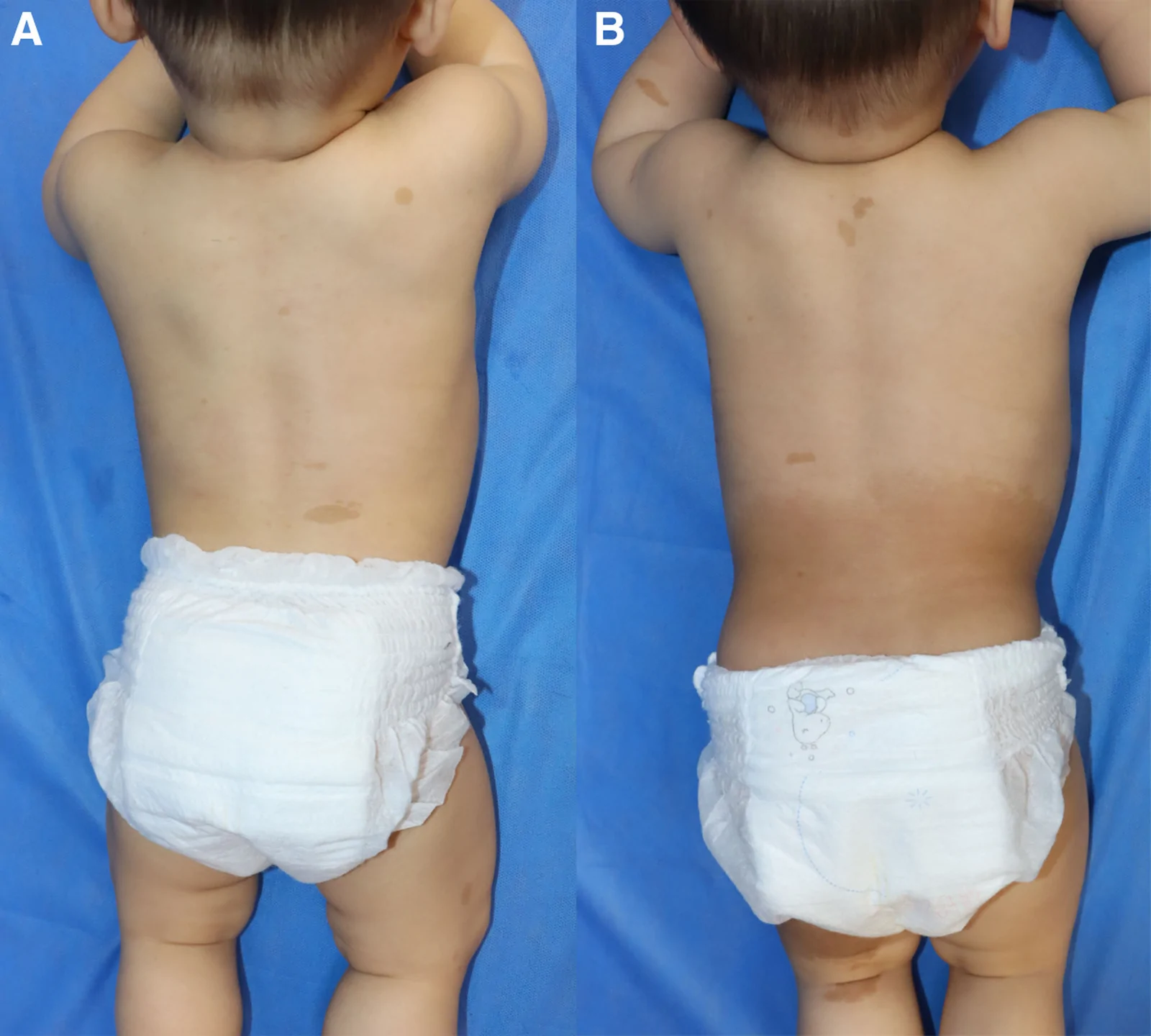
Dorsal skin of twin infants showing CALMs of varying sizes on the trunk, thighs, and limbs. (A) Twin A: scattered CALMs on the back and thighs. (B) Twin B: larger CALM on the lumbar region, with additional scattered CALMs on the back and thighs. CALMs = café-au-lait macules.

**Figure 3.**
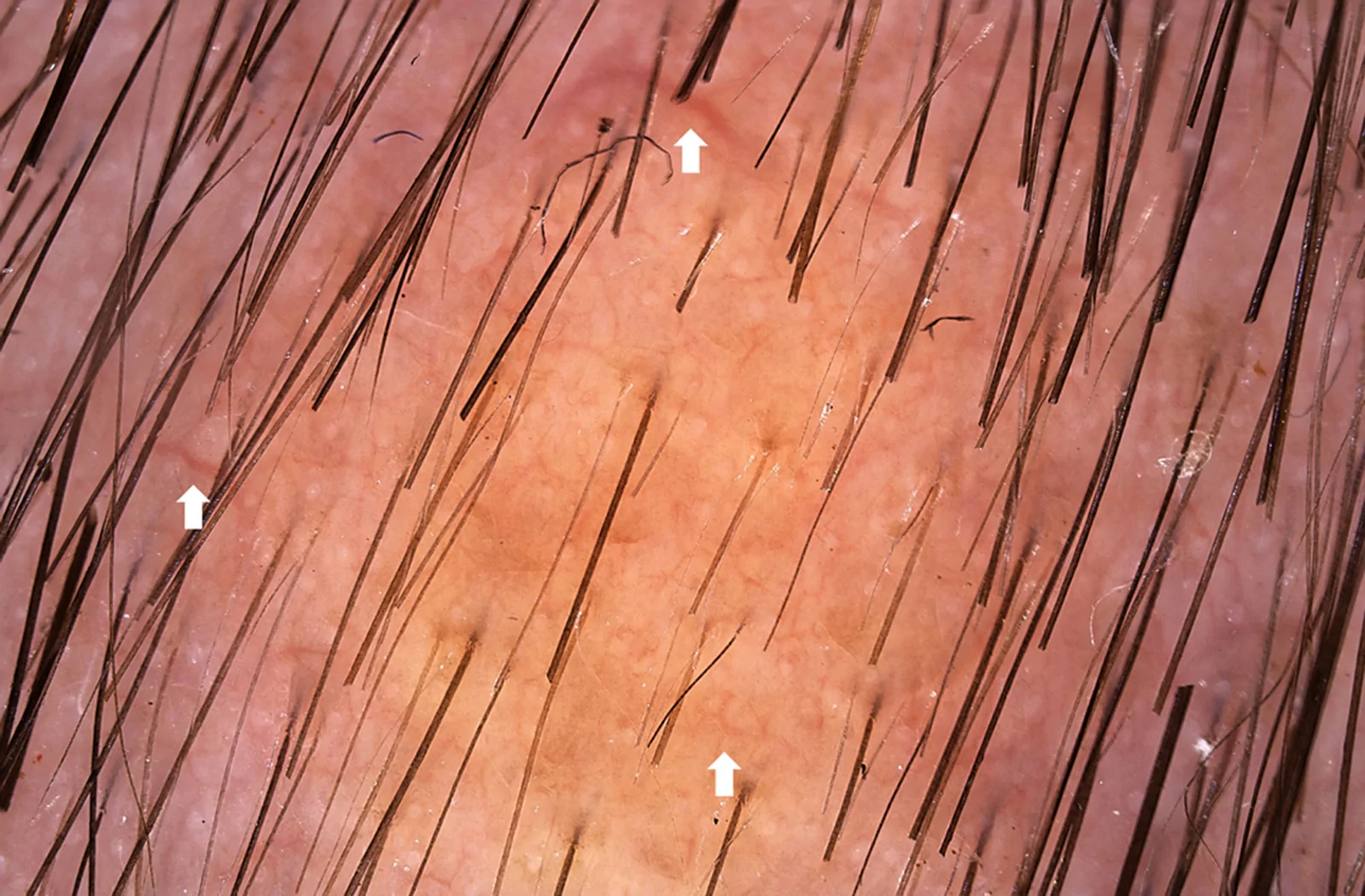
Dermoscopic view showing orange-yellow background with linear branched vessels (white arrows).

**Figure 4.**
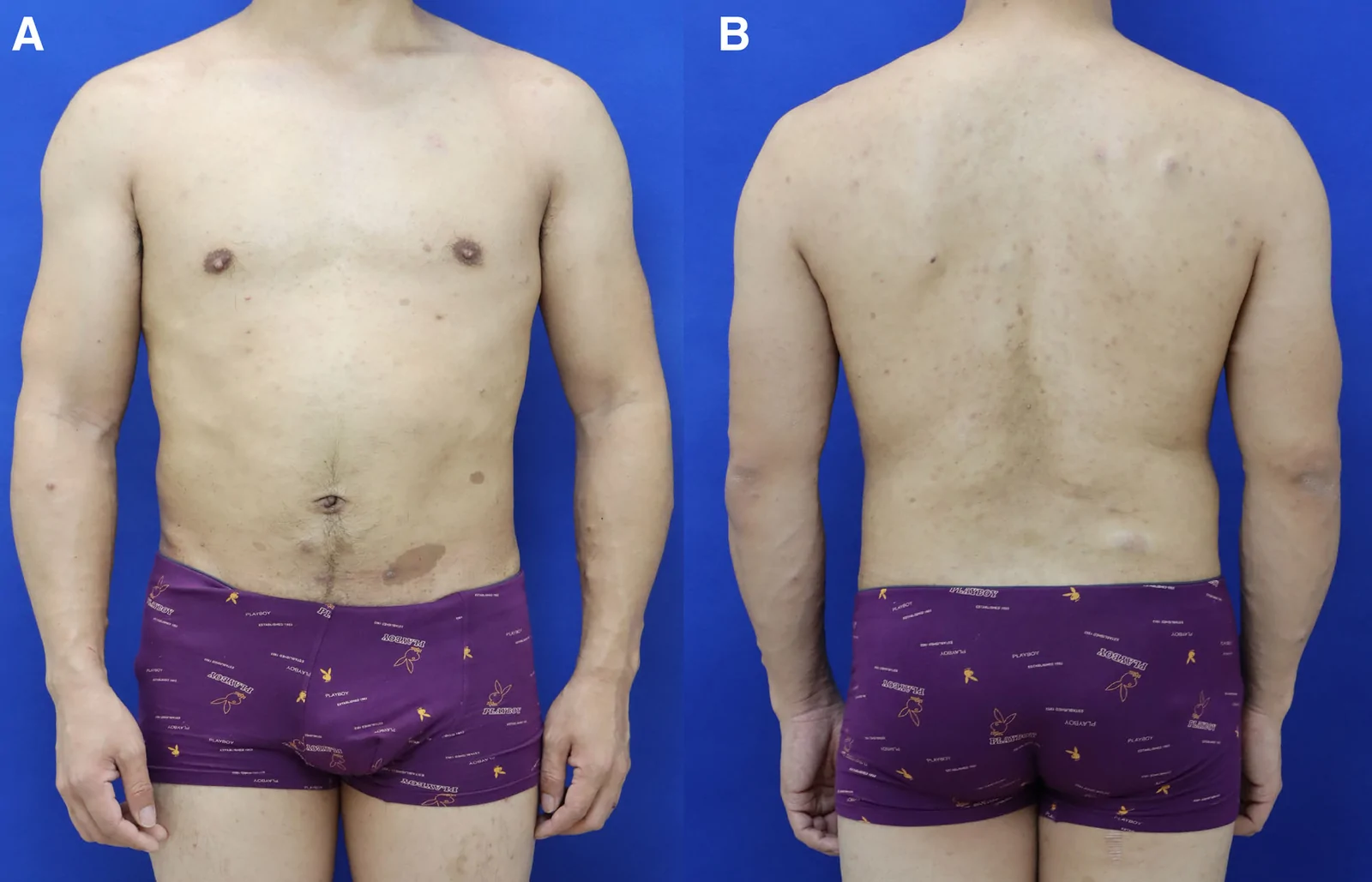
The twins’ father with multiple CALMs and subcutaneous nodules on the trunk and limbs. (A) Frontal view: prominent CALM on the lower abdomen. (B) Dorsal view: CALMs and subcutaneous nodules, consistent with NF1. CALMs = café-au-lait macules, NF1 = neurofibromatosis type 1.

Two 14-month-old twin infants presented with multiple yellow papules on their scalps, first noted 2 months prior. They were delivered by cesarean section at 38 weeks of gestation and have since demonstrated normal growth and development. Physical examination revealed several well-demarcated, yellow, round, firm papules, measuring 2 to 5 mm in diameter on their scalps (Fig. 1). Additionally, both twins had 10 to 20 CALMs on the trunk and limbs, with the largest lesion measuring over 15 cm, extending from the waist to the thigh (Fig. 2). Blood tests, including routine blood work, lipid profile, and liver and kidney function, were all within normal limits. Ophthalmologic screening and brain magnetic resonance imaging (MRI) showed no significant abnormalities. Dermoscopic examination of the yellow papules revealed multiple polymorphous vessels on a yellow-orange background, surrounded by erythema (Fig. 3). Genetic testing identified a mutation involving duplication of exon 2 in the NF1 gene. Further family history revealed that the father had multiple CALMs and cutaneous neurofibromas (Fig. 4), the latter confirmed by a prior biopsy. He exhibited no other NF1-related complications, such as hypertension, seizures, tumors, ocular abnormalities, or skeletal deformities. No similar findings or significant illnesses were reported in his parents or siblings. Genetic testing confirmed that the father carried the same NF1 exon 2 duplication identified in the twins, consistent with an autosomal dominant inheritance pattern. Based on the clinical history, physical examination, dermoscopic findings, laboratory results, and genetic testing, the diagnoses of JXG and NF1 were confirmed. The family was provided with a detailed explanation of the conditions, emphasizing the self-limiting nature of the JXG. Regular follow-up visits were recommended to monitor for potential age-related manifestations of NF1, with annual assessments including skin examination, ophthalmologic evaluation, growth and skeletal monitoring, blood pressure measurement, neurological and developmental screening, and imaging for plexiform neurofibromas (PNF) when indicated.^[3]^

At the 1-year follow-up, the younger twin developed swelling in the left gluteal region. Pelvic and abdominal MRI revealed an irregular mass extending from the posterior thigh to the left para-aortic region, exhibiting nodular and cord-like features with T1 hypointensity, heterogeneous T2 signal, and high intensity on fat-suppressed T2 sequences. The lesion involved adjacent muscles, fascial planes, and subcutaneous fat and extended through the intervertebral foramen into the spinal canal. These imaging characteristics were consistent with PNF, which was confirmed by biopsy. Selumetinib therapy is planned for this patient. In contrast, the elder twin remained asymptomatic. Full spinal and cranial MRI revealed no significant abnormalities apart from a small subcalvarial nodule in the parietal bone, likely of vascular origin, and mild mucosal thickening in the ethmoid and maxillary sinuses. No evidence of PNF or neurologic involvement was observed, and close clinical monitoring is ongoing.

## 3. Discussion

JXG is a rare, benign, self-limiting proliferative disorder characterized by the accumulation of non-Langerhans cell histiocytes.^[8]^ While JXG primarily affects infants and young children, cases of adult onset have also been reported.^[9]^ Clinically, JXG typically manifests as solitary or multiple asymptomatic papules or nodules, which are most commonly located on the head, neck, upper torso, or extremities. The lesions usually measure between 0.5 and 2.0 cm in diameter. Initially, they appear pink, gradually transitioning to a yellow-brown color, and may sometimes show surface telangiectasia.^[4]^ Dermoscopy often reveals the characteristic “setting sun” appearance, with an orange-yellow background and paler yellow spots.^[10]^ The diagnosis of JXG is mainly clinical, although biopsy may be performed when needed for confirmation. Histopathological examination typically reveals atypical histiocytes, foam cells, and scattered eosinophils and lymphocytes. In about 85% of cases, Touton giant cells are observed. Immunohistochemistry typically shows positivity for CD68, factor XIIIa, and CD4, while S-100 is negative.^[11]^ Most cases of JXG resolve spontaneously within months to years, although some may leave mild atrophic scars.^[12]^ For solitary cutaneous lesions, observation or simple surgical excision is usually sufficient.^[13]^

JXG involving 1 or more extracutaneous or visceral sites is classified as systemic juvenile xanthogranuloma (SJXG), a rare clinical entity accounting for ~5% of cases in a review of 174 patients. Reported extracutaneous sites include the eyes, oropharynx, lungs, liver, spleen, subcutaneous soft tissue, muscle, heart, kidneys, retroperitoneum, ribs, and central nervous system (CNS), with ocular involvement being the most commonly affected site.^[14]^ Compared with solitary cutaneous JXG, SJXG is associated with a poorer prognosis and is frequently complicated by severe conditions such as liver failure, sepsis, coagulopathy, and electrolyte disturbances.^[15]^ Studies indicate that an increased number of cutaneous lesions and a younger age at diagnosis are associated with a higher risk of systemic involvement.^[10]^ These findings highlight the importance of screening for visceral involvement in infants with multiple congenital cutaneous JXG lesions. Abdominal ultrasound should be incorporated as a routine diagnostic procedure in these patients to enable early detection and management of systemic disease. Treatment strategies for SJXG vary depending on the extent and location of organ involvement. A recent systematic review of 159 SJXG cases reported the use of chemotherapy in 41% of patients, surgery in 40%, radiotherapy in 8%, and hematopoietic stem cell transplantation in 2.5%.^[16]^ Among those with multiple extracutaneous lesions, the most commonly used first-line regimen was prednisolone combined with vinblastine, although this did not significantly improve overall survival compared with untreated patients. Surgery was frequently used for isolated ocular or CNS involvement. Radiotherapy served primarily as an adjunct for refractory or life-threatening cases. Hematopoietic stem cell transplantation, though rarely employed, has shown success in select patients unresponsive to systemic chemotherapy, with some achieving durable remission.

JXG has been associated with several other conditions, including NF1 and juvenile myelomonocytic leukemia (JMML).^[10]^ In NF1 patients, JXG may serve as an early manifestation of the disease, especially in children who present with CALMs but lack other characteristic features of NF1.^[5]^ Importantly, the risk of developing JMML is significantly higher in patients with both NF1 and JXG. Some studies have shown that NF1 patients with JXG have a 20- to 30-fold increased risk of developing JMML compared with those with NF1 alone.^[17]^ A retrospective study of 67 NF1 patients with JXG found that 12 (18%) were diagnosed with JMML, with JXG being diagnosed prior to JMML in all cases.^[10]^ However, other investigations have yielded conflicting results. For instance, a retrospective case-control study of 739 NF1 patients by Liy-Wong et al did not demonstrate a statistically significant association between JXG and malignancy (odds ratio = 1.5, 95% confidence interval: 0.35–6.6, *P* = .56).^[18]^ These discrepancies may reflect differences in sample size, methodology, or diagnostic criteria. While a biological link between NF1, JXG, and JMML remains plausible (especially in cases of early-onset or multiple JXG), current evidence does not support routine malignancy screening based solely on the presence of JXG in NF1 patients.

The co-occurrence of NF1 and JXG has raised interest in potential shared mechanisms. Genetic studies have identified mutations in Ras/MAPK pathway-related genes, including NF1, PTPN11, KRAS, and NRAS, in sporadic JXG cases, suggesting convergence via pathway dysregulation.^[17]^ Therapeutically, MEK inhibitors (e.g., selumetinib, cobimetinib, trametinib) target this pathway and are effective in NF1-related tumors. These agents also benefit other non-Langerhans cell histiocytoses with biological similarities to JXG, such as Erdheim-Chester disease, Rosai-Dorfman-Destombes disease, and progressive nodular histiocytosis.^[19–22]^ As shown in Table 1,^[1–3,16,23–31]^ NF1 and JXG share overlapping systemic involvement, including the eyes, lungs, liver, skeleton, and CNS, supporting potential pathophysiological links.

**Table 1:** Comparison of systemic involvement in JXG and NF1.

Systemic involvement	JXG	NF1
Ocular	Iris involvement is most common: asymptomatic iris tumors, glaucoma, hyphema, uveitis signs, heterochromia; also eyelid/orbit involvement.^[1]^	Two or more iris hamartomas (Lisch nodules).^[3]^
Pulmonary	Multiple round or nodular opacities mimicking metastases^[1]^; multifocal pulmonary parenchymal or interstitial infiltration.^[16]^	Diffuse lung disease with upper-lobe blebs, bullae, and cysts, and basilar fibrosis.^[23]^
Hepatosplenic/hematologic	Hepatomegaly, hyperbilirubinemia, liver failure; splenomegaly; anemia, thrombocytopenia; may also present with jaundice, elevated liver enzymes, and nonspecific imaging findings.^[1,16,24]^	Infiltration by plexiform neurofibromas may lead to liver dysfunction.^[25,26]^
Skeletal	Solitary osteolytic lesions in axial bones (ribs, skull, spine, pelvis); adjacent joints spared.^[16,27–31]^	Scoliosis, macrocephaly, prominent brow, long bone dysplasia, pseudoarthrosis, pectus excavatum, short stature.^[2]^
Nervous system (central and peripheral)	Brain, dura, ventricles, spinal cord involvement; diabetes insipidus, polyuria, polydipsia, paraplegia.^[16]^	Optic gliomas (low-grade); plexiform neurofibromas, malignant peripheral nerve sheath tumors.^[2]^

To our knowledge, this is the first reported case of monozygotic twins simultaneously diagnosed with both NF1 and JXG. Such a rare presentation may indicate shared genetic susceptibility and suggests that JXG is not always sporadic in NF1. Future studies should explore how germline NF1 mutations influence histiocytic proliferation, using multi-omics approaches such as transcriptomics, epigenetics, and mosaicism analysis. International registries and multicenter studies could further support evaluation of targeted therapies in SJXG.

## 4. Conclusion

This case report underscores the potential association between JXG and NF1, particularly in infants exhibiting CALMs without other characteristic features of NF1. These findings suggest that JXG may represent an early manifestation of NF1, highlighting the importance of thorough clinical evaluation and genetic testing in such cases. The underlying pathological mechanisms shared between JXG and NF1 may involve dysregulation of the Ras/MAPK signaling pathway. Future studies should aim to further elucidate the molecular mechanisms connecting these 2 conditions and explore the potential therapeutic role of MEK inhibitors in the management of JXG.

## Acknowledgments

The authors would like to express their gratitude to the patient for their cooperation and support.

## Author contributions

**Writing – original draft:** Lingxi Liu.

**Writing – review & editing:** Lingxi Liu, Chunyan Mao.
